# Supplementary material for: Molecular basis of ParA ATPase activation by the CTPase ParB during bacterial chromosome segregation
Source: Nat Commun. 2025 Sep 25;16:8428. doi: 10.1038/s41467-025-63976-0 (PMC12462528; doi:10.1038/s41467-025-63976-0)
Supplement: Supplementary file 1 — Supplementary Information [file 41467_2025_63976_MOESM1_ESM.pdf]

Supplementary Information

Molecular basis of ParA ATPase activation by the CTPase ParB during bacterial chromosome segregation

Lucas Schnabel, Manuel Osorio-Valeriano, Cecilia Perez-Borrajero, Wieland Steinchen, Christopher-Nils Mais, Bernd Simon, Maria Thamm, Janosch Hennig, Gert Bange, Martin Thanbichler

SUPPLEMENTARY FIGURES

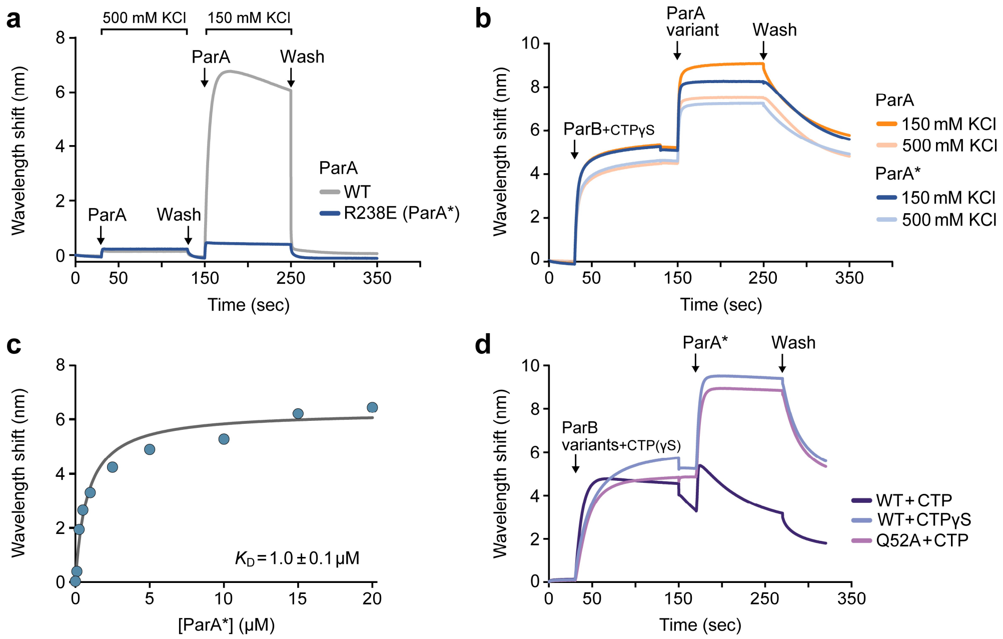

Supplementary Figure 1. Model partition complexes offer a robust tool to study the ParA-ParB interaction. (a) Biolayer interferometry (BLI) analysis investigating the effect of elevated salt concentrations on the non-specific DNA-binding activity of ParA. Double-biotinylated DNA fragments containing a central *M. xanthus parS* site were immobilized on a biosensor and probed with wild-type ParA or its DNA-binding-deficient variant ParA-R238E (ParA*) (10 µM) in a buffer containing 500 mM KCl. After the association phase, the biosensor was transferred into protein-free buffer to removed bound protein (Wash). Subsequently, an analogous binding assay was performed in a buffer containing only 150 mM KCl. **(b)** BLI analysis showing robust binding of ParA to DNA-bound ParB dimers in high-salt conditions. ParB was loaded onto a closed *parS*-containing DNA fragment (see **Figure 1a**) probed with wild-type ParA or ParA-R238E (ParA*) (10 µM) in a buffer containing ATP and CTP (1 mM each) and either 150 mM or 500 mM KCl. **(c)** Determination of the affinity of ParA-R238E (ParA*) for DNA-bound ParB dimers. The wavelength shift values reached at the end of the association phase in the experiments described in **Figure 1b** were plotted against the corresponding ParA concentrations and fitted to a non-cooperative one-site specific-binding model. The graph shows the results of a representtative experiment. The K_D value given in the graph represents the mean (± SD) of three independent replicates. **(d)** BLI analysis of the interaction of ParA-R238E (ParA*) with ParB in different nucleotide states. The indicated ParB proteins were loaded onto closed *parS*-containing DNA (see **Figure 1a**) and probed with ParA* (5 µM) in the presence of ATP (1 mM).

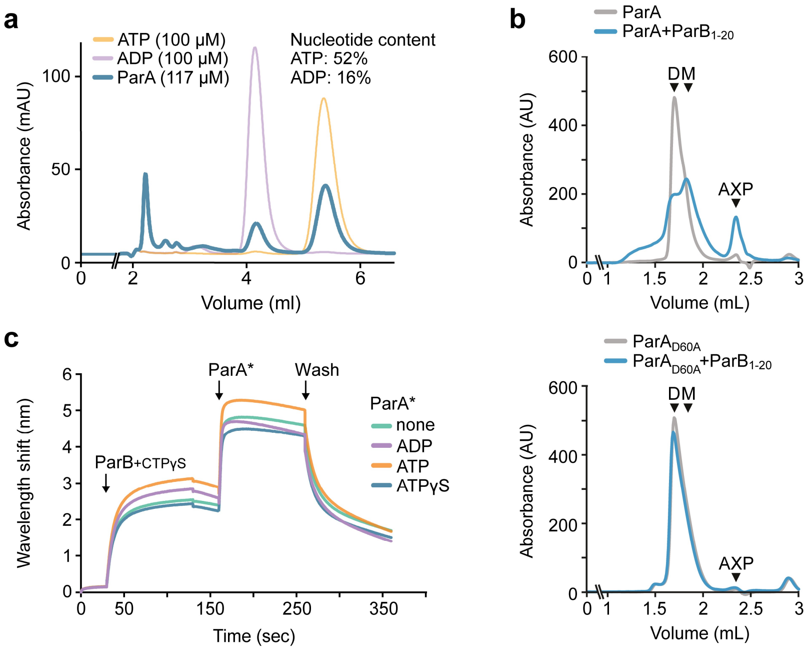

Supplementary Figure 2. ***M. xanthus* ParA is purified in the ATP-bound state. (a)** Nucleotide content analysis of purified ParA. ParA (117 µM) was denatured, and the released nucleotides were separated by high-performance liquid chromatography (HPLC). Standard solutions of ATP and ADP (100 µM each) were analyzed as a reference. Nucleotides were detected at a wavelength of 260 nm. The relative nucleotide content of ParA is indicated in the graph. The graph shows a representative experiment, which was performed twice with similar results. **(b)** Size-exclusion chromatographic analysis of the oligomerization state of purified ParA and ParA-D60A. ParA or the ATPase-deficient variant ParA-D60A (75 µM) were incubated for 2 min in ATP-free buffer the absence or presence of the ATPase-stimulating $ParB_{1-20}$ peptide (1.2 mM). Subsequently, the mixtures were separated by size-exclusion chromatography. Protein monomers (M) and dimers (D) as well as free nucleotides (AXP) were detected in the eluate photometrically at 280 nm. **(c)** BLI analysis investigating the dependence of the interaction of purified ParA with ParB on the adenosine nucleotide supplied in the reaction buffer. ParB was loaded onto a closed *parS*-containing DNA fragment (see **Figure 1a**) and probed with purified ParA-R238E (ParA*) in reaction buffers containing ADP, ATP or ATPγS or no additional nucleotide.

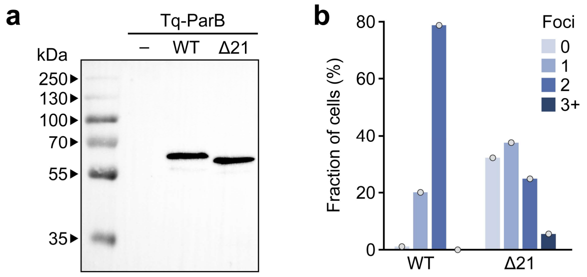

Supplementary Figure 3. ***In vivo*** **analysis of ParB variants. (a)** Immunoblot analysis of cells producing Tq-ParB (MO072) or Tq-ParBΔ21 (LS007) in place of wild-type ParB, as analyzed in **Figure 1e**. Proteins were detected with an anti-GFP antibody. A Δ*parB* mutant producing untagged ParB under the control of an inducible promoter (SA4269) was used as a negative control (-). **(b)** Quantification of the number of distinct fluorescent foci in cells producing Tq-ParB (MO072, n=358 cells) or Tq-ParBΔ21 (LS007, n=326 cells) in place of wild-type ParB, taken from the cultures analyzed in **Figure 1e**.

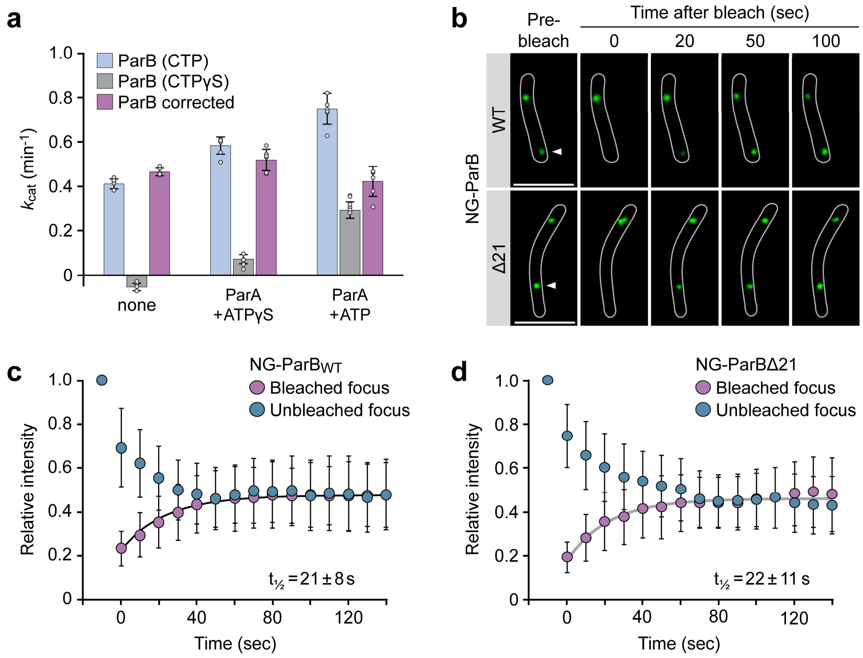

Supplementary Figure 4. ParA binding has no effect on the CTPase activity of ParB *in vitro* or its localization dynamics *in vivo*. (a) Effect of ParA on the CTPase activity of ParB. ParB (5 µM) was incubated alone or with ParA (5 µM) in the presence of ATP or ATPγS (1 mM) in a buffer containing CTP or CTPγS (1 mM), salmon sperm DNA (100 µg/mL) and a *parS*-containing DNA stem-loop (150 nM). The rate of nucleotide hydrolysis was determined with a coupled enzyme assay, measuring the rate of phosphate release as a proxy. To determine the turnover rates for ParB under the given conditions, the rates measured for the CTP-containing reactions were corrected for the ATPase activity of ParA, measured in reactions containing the poorly hydrolysable CTP analog CTPγS. The data represent the mean of six independent replicates (± SD). **(b)** Fluorescence-recovery-after-photobleaching (FRAP) analysis of the effect of the ParA-ParB interaction on the mobility of ParB in *M. xanthus* cells. Cells were depleted of the wild-type ParB protein and induced to produce mNeonGreen (NG)-ParB (LS014) or NG-ParBΔ21 (LS015). In S-phase cells containing two clearly distinguishable partition complexes, one of the foci was bleached with a short laser pulse, and the recovery of the fluorescence signal was followed over time. The panels show fluorescence images of representative cells before bleaching and at the indicated times after application of the laser pulse. The bleached region is indicated by arrowheads. Scale bar: 3 µm. **(c,d)** Quantification of the kinetics of fluorescence recovery in the experiments described in panel b. The average relative integrated intensities of the bleached and unbleached partition complex (± SD) are plotted as a function of time for cells producing (C) NG-ParB (n= 30 cells) or (D) NG-ParBΔ21 (n=35 cells). The recovery half-times (± SD) indicated in the graph were determined by fitting of the data to a single-exponential function.

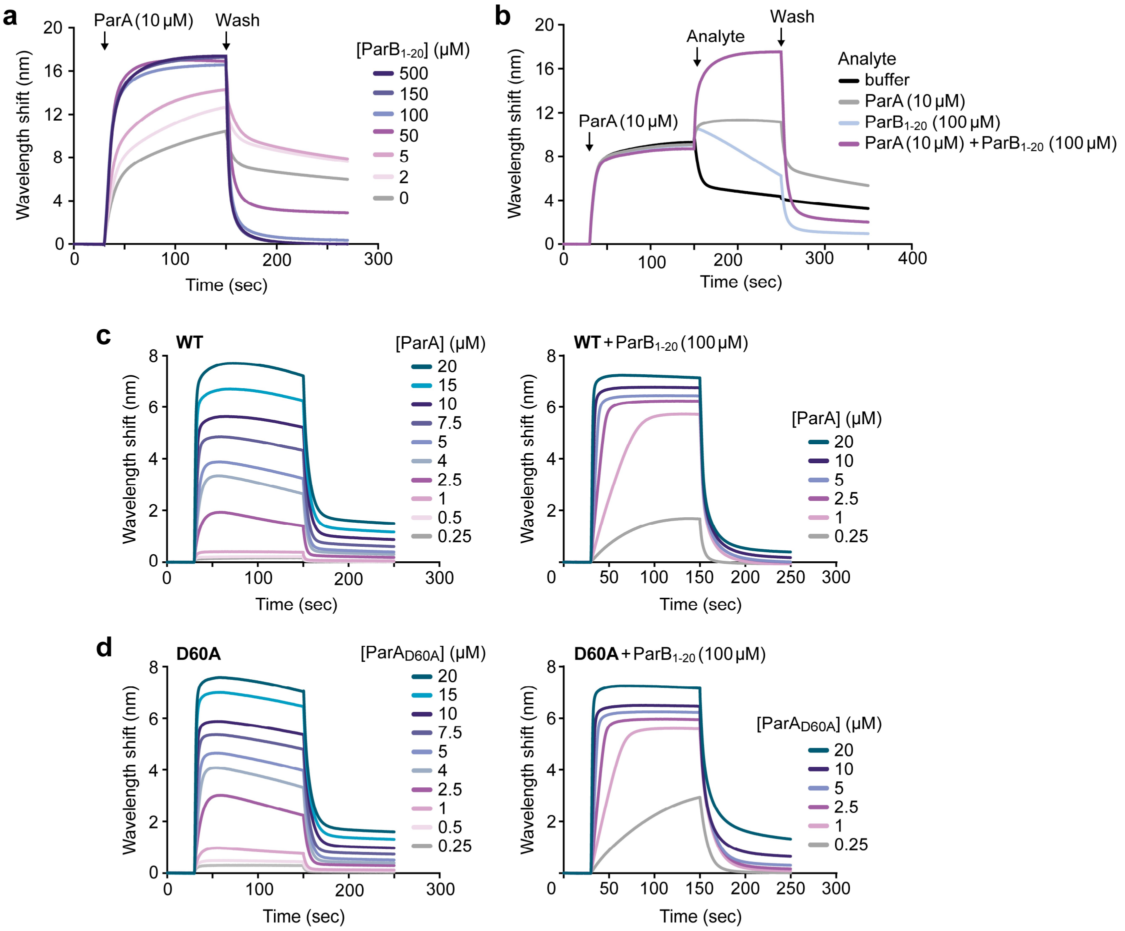

Supplementary Figure 5. ParB$_{1-20}$ stimulates the DNA-binding activity of ParA. (a) BLI analysis of the interaction of ParA with DNA in the presence of increasing concentrations of ParB$_{1-20}$. Streptavidin-coated biosensors carrying a closed double-biotinylated DNA fragment (234 bp) were probed with ParA (10 µM) in the presence of ATP and the indicated concentrations of ParB$_{1-20}$ peptide. At the end of the association phase, the biosensor was transferred into protein- and nucleotide-free buffer to monitor the dissociation reactions (Wash). **(b)** BLI analysis of the effect of ParB$_{1-20}$ on DNA-bound ParA. A biosensor carrying a closed double-biotinylated DNA fragment (234 bp) was incubated with ParA in the presence of ATP (1 mM). Subsequently, it was transferred into a buffer containing ATP (1 mM) and either no protein, ParA (10 µM), ParB$_{1-20}$ (100 µM) or both ParA and ParB as analytes. After monitoring the resulting change in the degree of ParA binding, the biosensor was transferred into protein- and nucleotide-free buffer to follow the dissociation reaction. **(c,d)** BLI analysis investigating the effect of ParB$_{1-20}$ on the DNA-binding activity of (c) ParA or (d) its ATPase-deficient variant ParA-D60A at different ParA concentrations. Streptavidin-coated biosensors carrying a closed double-biotinylated DNA fragment (234 bp) were probed with ParA or ParA-D60A (10 µM) in the presence of ATP (1 mM) and the indicated concentrations of ParB$_{1-20}$ peptide.

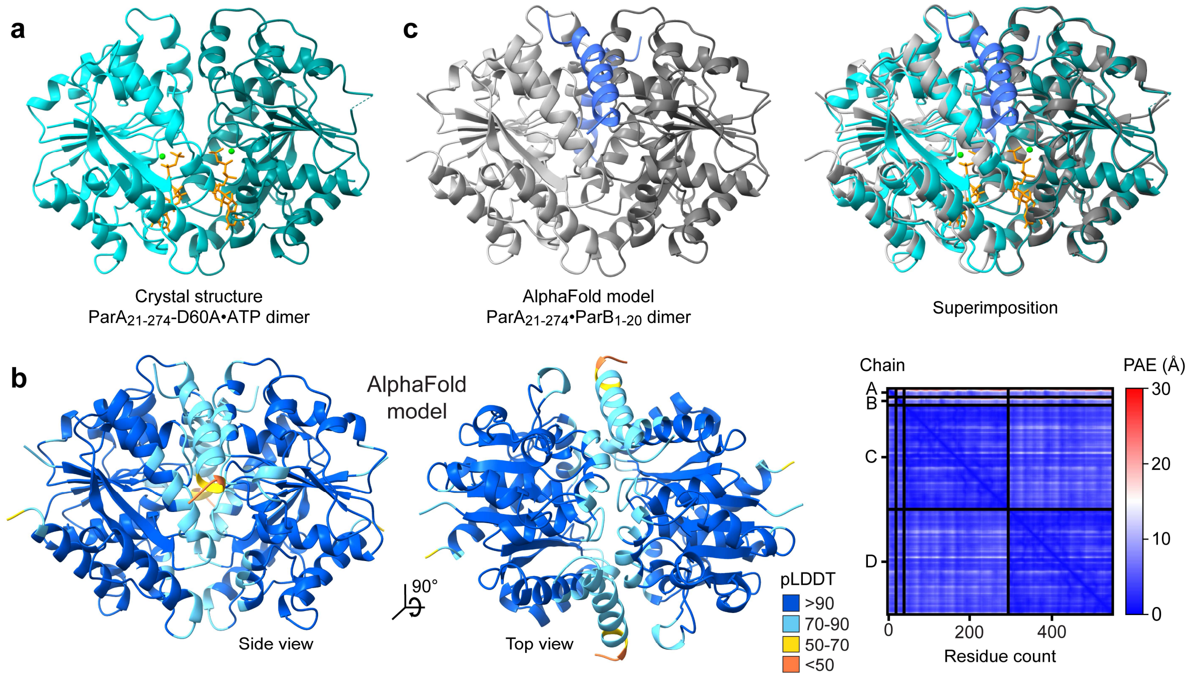

Supplementary Figure 6. Structural analysis of ParA•ATP dimers in complex with the ParB$_{1-20}$ peptide. (a) Crystal structure of the ParA$_{21-274}$-D60A•ATP dimer, shown in cartoon representation. The two subunits are displayed in cyan and teal. The two ATP molecules (orange) and Mg^{2+} ions (green) are highlighted. **(b)** Predicted structure of an *M. xanthus* ParA$_{21-274}$ dimer in complex with two molecules of ParB$_{1-20}$, determined with Alphafold-Multimer [1] and shown in cartoon representation. The structures are colored by pLDDT. The whole-structure pLDDT value is 93.1, and the ipTM value for the ternary complex is 0.874. The heatmap shows the predicted aligned error (PAE) for pairs of residues in the two ParB$_{1-20}$ (A and B) and ParA (C and D) molecules. **(c)** Superimposition of the predicted structure of a ParA$_{21-274}$ dimer in complex with two molecules of ParB$_{1-20}$ onto the crystal structure of the ParA$_{21-274}$-D60A•ATP dimer from panel a. The two subunits of the modeled ternary complex are shown in dark and light grey, and the ParB$_{1-20}$ molecules are highlighted in blue.

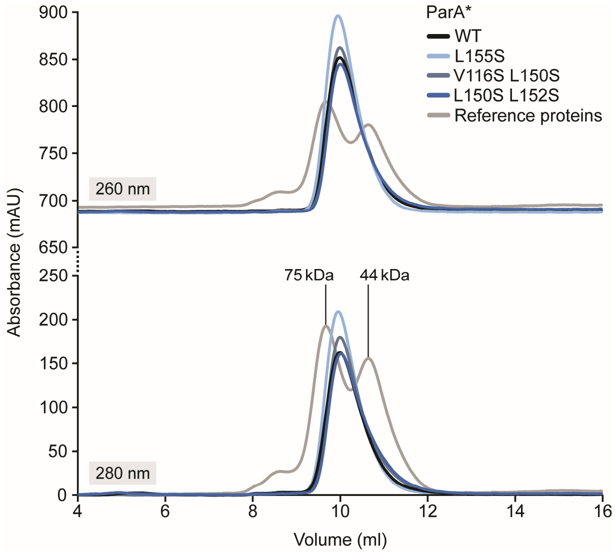

Supplementary Figure 7. Size-exclusion chromatography analysis confirms the ability of mutant ParA variants to form ATP-bound dimers. Wild-type ParA* and the indicated mutant ParA* variants (2.5 mg/ml) were applied to a Superdex 75 3.2/300 size-exclusion column. The eluate was analyzed photometrically at wavelengths of 260 nm and 280 nm, corresponding to the absorption maxima of ATP and protein, respectively. Conalbumin (75 kDa) and ovalbumin (44 kDa) were analyzed as a reference. The molecular weight of a His_6-tagged ParA monomer is ~33 kDa. The peak positions observed show that all ParA* variants formed stable dimers. Their increased absorption at 260 nm relative to the reference proteins confirms their association with nucleotides.

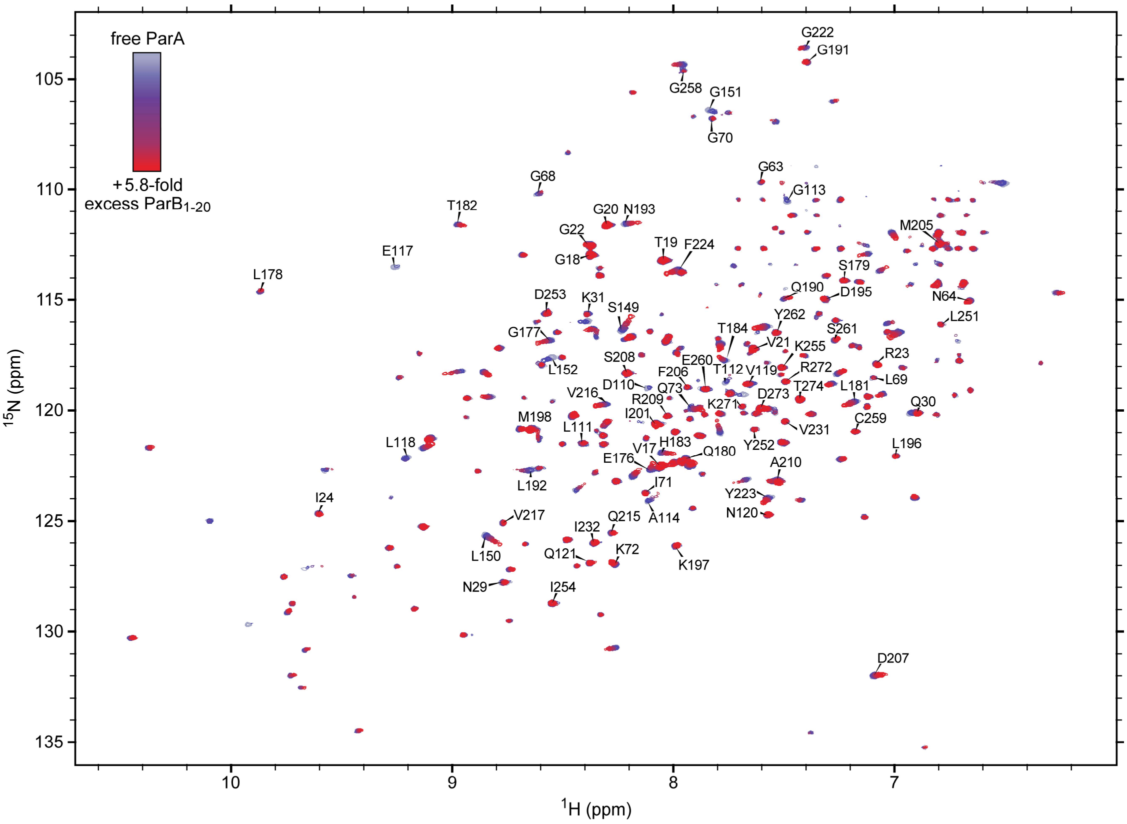

Supplementary Figure 8. NMR analysis reveals the binding site of $ParB_{1-20}$ on the $ParA_{21-274}$-D60A dimer. Shown are chemical shift perturbations (CSPs) observed upon titration of isotopically labeled $ParA_{21-274}$-D60A dimers with increasing concentrations of unlabeled $ParB_{1-20}$ peptide in a two-dimensional ^1H-^{15}N heteronuclear single quantum correlation (HSQC) experiment. Assigned residues are indicated in the figure.

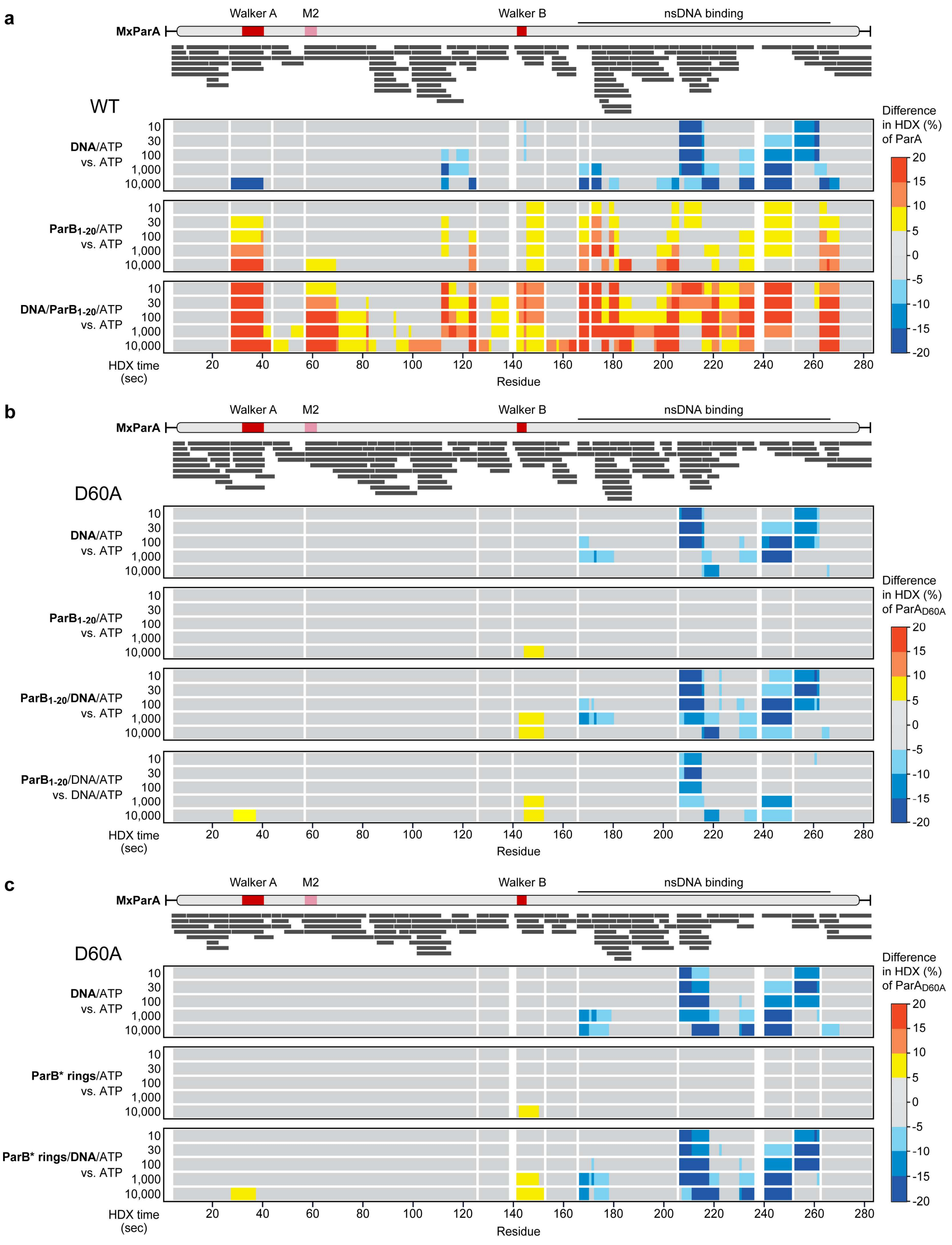

Supplementary Figure 9. HDX profiles of ParA and ParA-D60A dimers in the ligand-bound states. (a) Effect of DNA and $ParB_{1\text{-}20}$ on wild-type ParA dimers (see also **Supplementary Data 1**, Dataset 1). The heatmaps show the difference in HDX for ParA incubated with salmon sperm DNA (1 mg/mL), $ParB_{1\text{-}20}$ (1 mM) or both ligands compared to ParA incubated alone in deuterated buffer containing ATP (1 mM), mapped onto the amino acid sequence of ParA. The scheme on top shows the domain organization of *M. xanthus* ParA. Each black bar represents a ParA-derived peptide analyzed for its degree of deuterium incorporation. **(b)** Effect of DNA and $ParB_{1\text{-}20}$ on ParA-D60A dimers (see also **Supplementary Data 1**, Dataset 2). The heatmaps show the difference in HDX for ParA-D60A incubated with salmon sperm DNA (1 mg/mL), $ParB_{1\text{-}20}$ (1 mM) or both ligands compared to ParA-D60A incubated alone or with DNA in deuterated buffer containing ATP (1 mM), mapped onto the amino acid sequence of ParA. The scheme on top shows the domain organization of *M. xanthus* ParA. Each black bar represents a peptide derived from ParA-D60A analyzed for its degree of deuterium incorporation. **(c)** Effect of DNA and ParB rings on ParA-D60A dimers (see also **Supplementary Data 1**, Dataset 3). The heatmaps show the difference in HDX for ParA-D60A incubated with salmon sperm DNA (1 mg/mL), with ParB-Q52A (ParB*) (100 µM) plus a *parS*-containing DNA stem-loop (2.5 µM) or with all three components compared to ParA-D60A incubated alone in deuterated buffer containing ATP and CTP (1 mM each), mapped onto the amino acid sequence of ParA. The scheme on top shows the domain organization of *M. xanthus* ParA. Each black bar represents a peptide derived from ParA-D60A analyzed for its degree of deuterium incorporation. ParB-Q52A rings were pre-formed by incubation of the protein with the *parS*-containing DNA stem-loop and CTP for 30 min at ambient temperature and then mixed with the other components immediately prior to analysis.

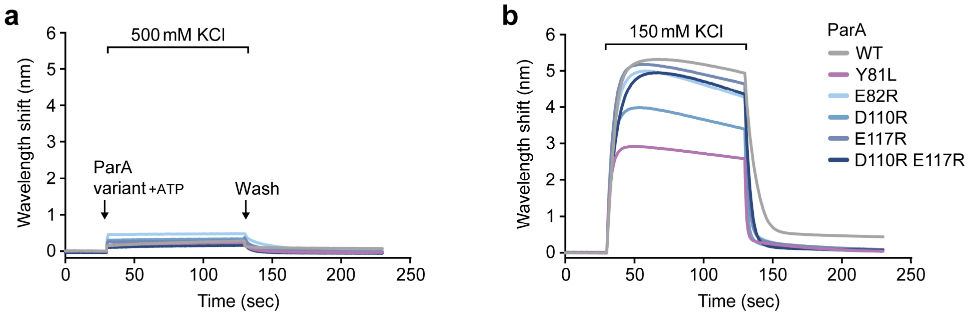

Supplementary Figure 10. Substitutions in the helix H4/H6/H7 region have at most mild effects on the DNA-binding activity of ParA. (a,b) BLI analysis investigating the DNA-binding activity of the indicated ParA variants in (A) high-salt (500 mM KCl) and (B) low-salt (150 mM KCl) conditions. Streptavidin-coated biosensors carrying a closed double-biotinylated DNA fragment (234 bp) were probed with ParA proteins (5 µM) in the presence of ATP (1 mM). At the end of the association phase, the biosensors were transferred into protein- and nucleotide-free low-salt buffer to monitor the dissociation reactions (Wash).

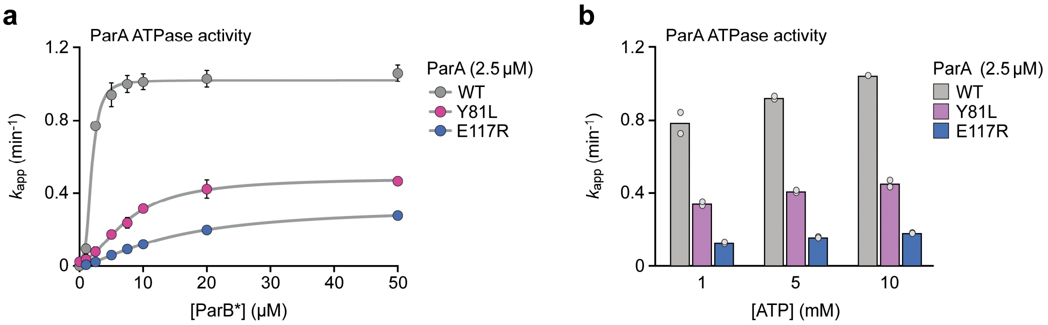

Supplementary Figure 11. ATPase activities of ParA variants with exchanges in the putative R13-binding region. (a) ATPase activities of wild-type ParA or the indicated mutant variants (2.5 µM) incubated with ATP (1 mM) and salmon sperm DNA (100 µg/ml) in the presence of different concentrations of ParB-Q52A (ParB*), closed by preincubation with CTP (1 mM) and a *parS*-containing DNA stem-loop (250 nM). Data represent the mean (± SD) of three independent replicates. The results were fitted to a Hill equation, as described for **Figure 1f**. **(b)** ATPase activities of wild-type ParA or the indicated mutant variants (2.5 µM) incubated with different concentrations of ATP in the presence of salmon sperm DNA (100 µg/ml) and ParB (10 µM), closed by preincubation with CTPγS (1 mM) and a *parS*-containing DNA stem-loop (250 nM). Data represent the mean of two independent replicates.

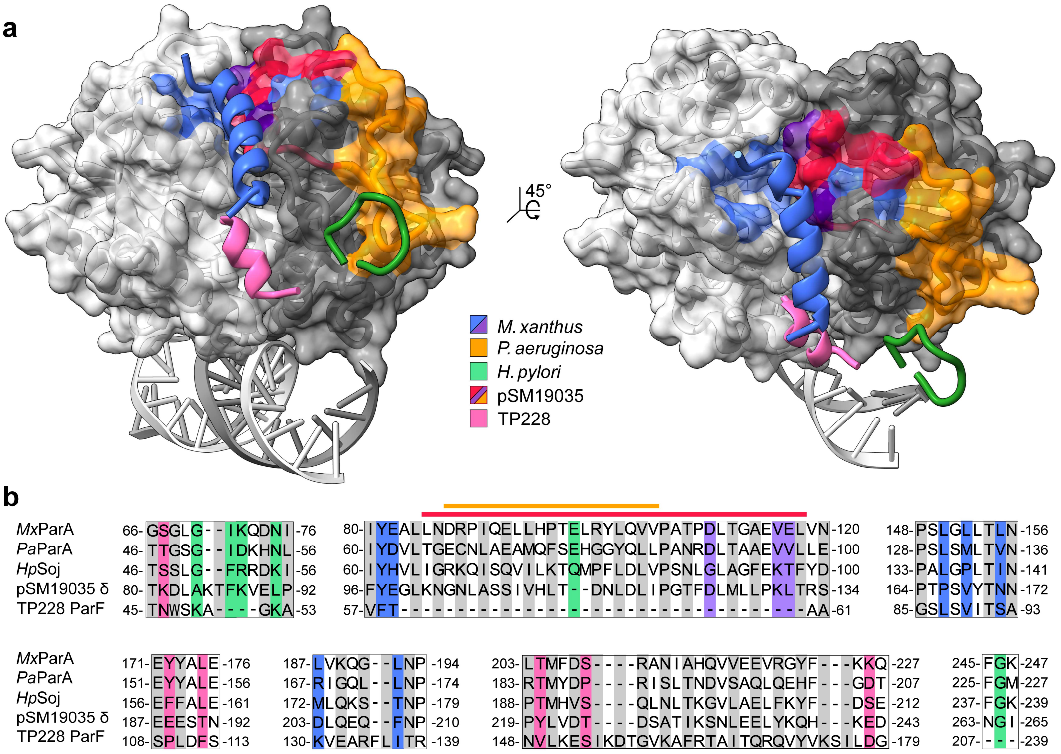

Supplementary Figure 12. Comparison of the ParB-binding site identified in this study with previously suggested binding sites. (a) ParB-binding sites suggested for different ParA orthologs, mapped onto the predicted structure of the *M. xanthus* $ParA_{21\text{-}274}$ dimer in complex with DNA, generated by superimposition of the crystal structure of the *M. xanthus* $ParA_{21\text{-}274}$-D60A•ATP dimer with the crystal structure of the DNA-bound *H. pylori* ParA-D41A•ADP dimer [2] (PDB: 6IUD). Shown are the N-terminal peptide of *M. xanthus* ParB and its interacting residues in *M. xanthus* ParA (blue/purple; this study), the location of a region of *P. aeruginosa* ParA required, directly or indirectly, for interaction with ParB in two-hybrid studies [3] (orange), the location of a short N-terminal peptide of ParB lacking part of the conserved ParA-binding motif on *H. pylori* ParA, as obtained by X-ray crystallography [4] (green), the location of a peptide of pSM19035 δ shown to reside in proximity of its bound ParB ortholog by chemical crosslinking studies [5] (red/purple/orange) and the location of a short N-terminal peptide of ParG on TP228 ParF identified by X-ray crystallography [6] (light red). **(b)** Mapping of the residues or regions implicated in ParB/ParG binding onto the amino acid sequences of the corresponding ParA proteins, compared in a multiple sequence alignment. Individual residues involved in the interactions are highlighted by colored backgrounds. Regions or peptides are indicated by colored lines on top of the alignment.

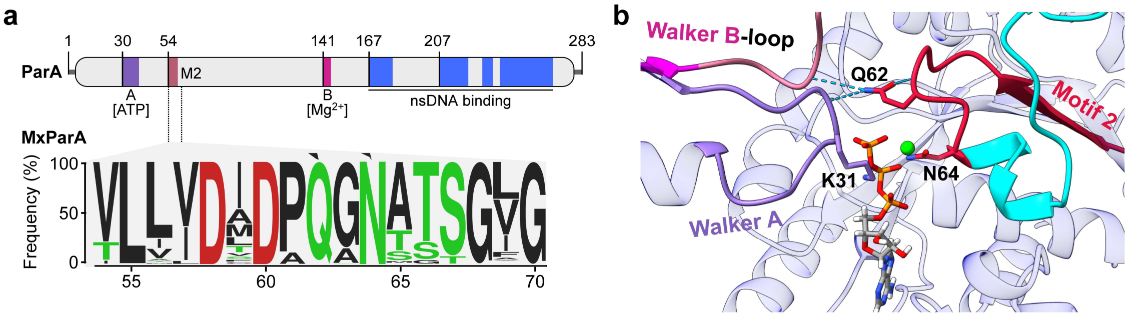

Supplementary Figure 13. Conserved interaction between the Motif 2 and Walker B regions of ParA. (a) Conservation of the Motif 2 region in ParA homologs. Shown are a schematic depicting the domain organization of *M. xanthus* ParA and a sequence logo showing the conservation of residues in the Motif 2 region (corresponding to residues 54-70 of *M. xanthus* ParA), based on an alignment of 3,800 ParA homologs obtained by protein BLAST analysis with *M. xanthus* ParA as a query. Residues are colored according to their physico-chemical properties (black: hydrophobic, red: negatively charged, green: polar). **(b)** Close-up of the catalytic center of *M. xanthus* ParA, based on the crystal structure of the His_6-$ParA_{21\text{-}274}$•ATP dimer. The Walker A loop, the Walker B-proximal loop and Motif 2 are highlighted. The conserved catalytic residues K31 and N64 as well as the highly conserved residue Q62 in Motif 2, which forms hydrogen bonds (dotted blue lines) with the backbone of the Walker B-proximal loop and the P-loop in the *trans*-subunit, are shown in stick representation.

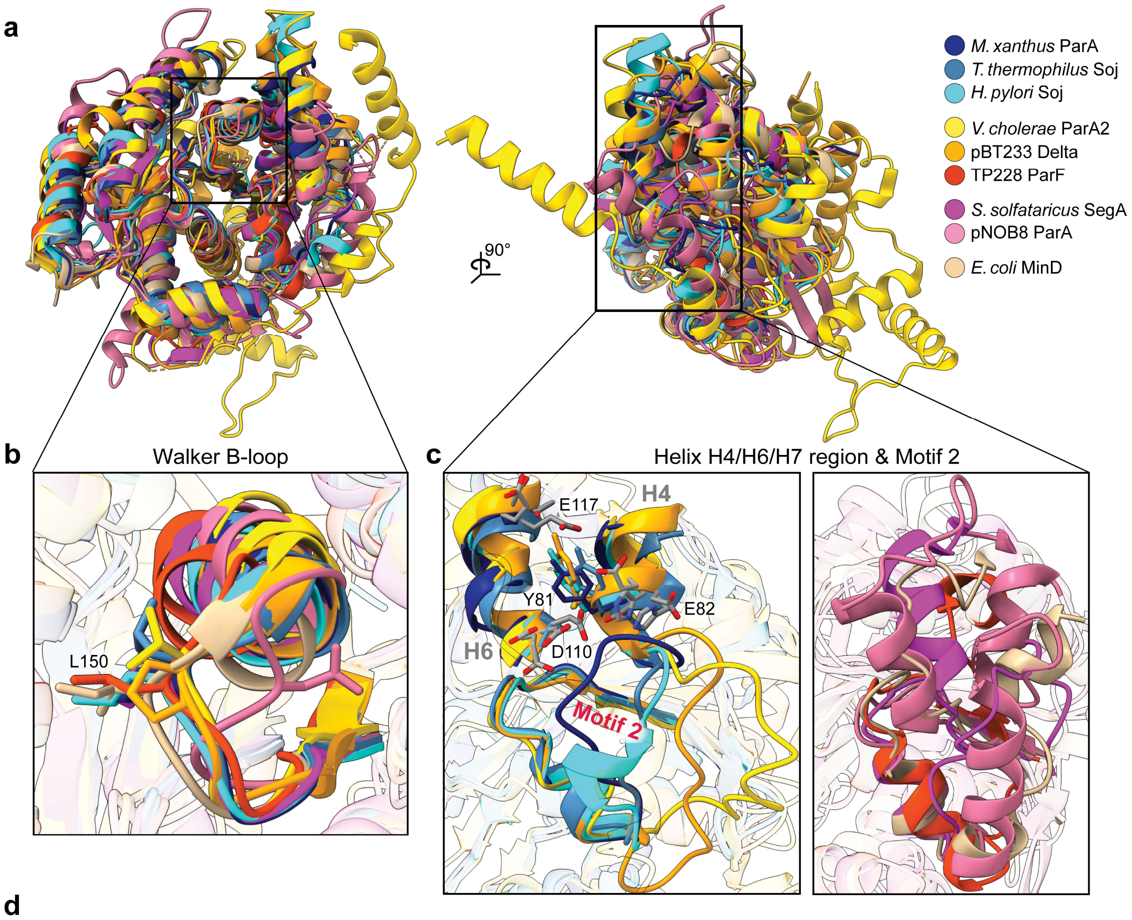

d

Chromosomal		Walker B-loop	Motif 2	H4/H6/H7	Amino acids 1-30
M. xanthus	ParA	hhhhDCPPS	hhhDMDPQGN	Y (EDE)	MVKADMQKRALGRGLSALIPQAGATGAGKG
T. thermophilus	Soj	hhhhDAPPS	hhhDLDPQAN	Y (HDE)	MSRKPSGLGRGLEALLPKTGAGVVRLPLAS
H. pylori	Soj	hhhhDSPPAL	hhhDFDPQGN	Y (HGT)	MAKNKVLGRGLADIFPEINEVYEQGLYERA
**Plasmid**					
V. cholerae chr2	ParA2	hhhhDTGPHV	hhhDLDPQGS	V (DEE)	MAIKTSELNAKLFGKADKRRATTPAEAQSA
pSM19035	Delta	hhhhDTVPTP	hhhDKDLQAT	Y (EDL)	MIVGNLGAQKAKRNDTPISAKKDIMGDKTV
TP228	ParF	hhhhDGAGSL	hhhDTDPQMS	(-)	MSLEKAHTSVKKMTFGENRDLERVVTAPVS
**Archaeal**					
S. solfataricus	SegA	hhhhDTPPNL	hhDLDPEGS	(-)	MSTIRIEMEKLGDLIPHEDILIDKVVNISF
pNOB8	ParA	hhhhDTAPVN	hhhDFDAQMS	(-)	MSKLKEYKEISIEEIKEVEEFKNFVPENNM
**Analogue**					
E. coli	MinD	hhhCDSPAGI	hhhDFDIGLRN	(-)	MALLDFFLSRKKNTANIAKERLQIIVAERR

Supplementary Figure 14. Conservation of the ParB binding site among members of the ParA/MinD ATPase family. (a) Superimposition of the crystal structures of *M. xanthus* ParA, *T. thermophilus* Soj (PDB: 2BEK) [7], *H. pylori* Soj (PDB: 6IUB) [2], *V. cholerae* ParA2 (PDB: 7NPD) [8], pSM19035 δ (PDB: 2OZE) [9], TP228 ParF (PDB: 4E09) [10], *S. solfataricus* SegA (PDB: 7DV3) [11], pNOB8 ParA (PDB: 5K5Z) [12] and *E. coli* MinD (PDB: 3Q9L) [13] monomers. The two overlays show a top view of the DNA-binding site, including the Walker B-proximal loop (left) and the region around Motif 2, including the Helix H4/H6/H7 region (right). **(b)** Magnified view of the Walker B-proximal loop from the indicated superimposition in panel a. Residues corresponding to L150 of *M. xanthus* ParA are displayed as sticks. **(c)** Magnified views of the helix H4/H6/H7 and Motif 2 regions from the indicated superimpositions in panel a. *M. xanthus* ParA, *T. thermophilus* Soj, *H. pylori* Soj, *V. cholerae* ParA2 and pSM19035 δ share similar structures in this region. For these proteins, a view of the helix H4/H6/H7 regions and a stick representation of the residues corresponding to Y81, E82, D110 and E117 of *M. xanthus* ParA are shown on the left. The H4/H6/H7 region is not conserved in TP228 ParF, *S. solfataricus* SegA, pNOB8 ParA and *E. coli* MinD. A superimposition of the regions surrounding Motif 2 in these proteins is shown on the right. **(F)** Overview of functionally important regions in ParA orthologs and their corresponding ATPase-stimulating proteins. Shown are the sequences constituting the Walker B-loop and Motif 2 as well as the amino acids corresponding to Y81, E82, D110 and E117 in the H4/H6/H7 region of *M. xanthus* ParA together with the first 30 amino acids of their corresponding ATPase stimulating proteins. Hydrophobic residues are denoted “h”, conserved ParA-binding sequences are highlighted in blue.

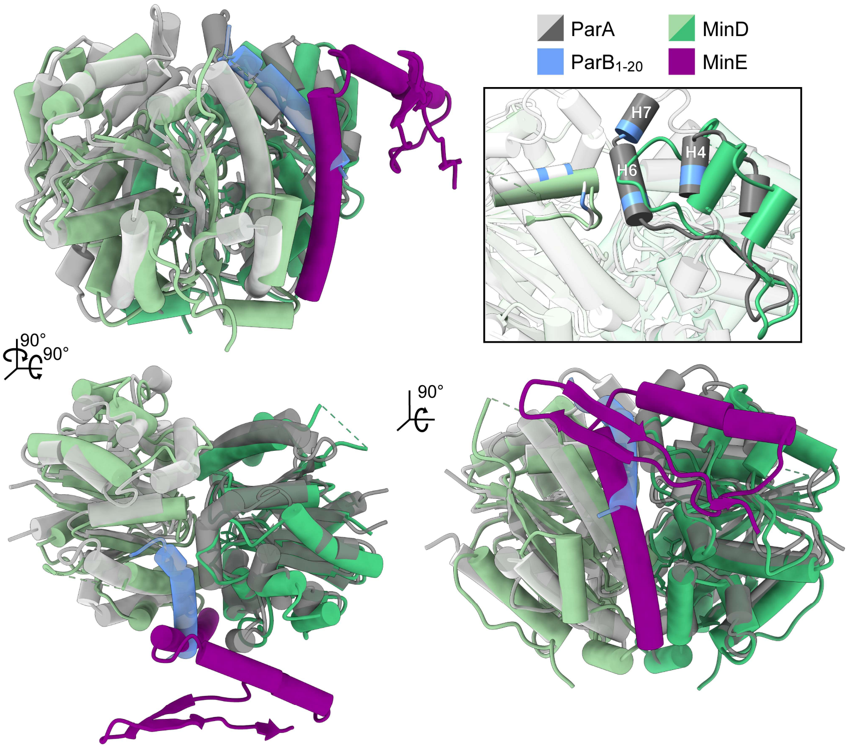

Supplementary Figure 15. Comparison of the $ParA_2$•$ParB_{1\text{-}20}$ and $MinD_2$•MinE complexes. Shown are superimpositions of the predicted structure of the *M. xanthus* ParA dimer in complex with the $ParB_{1\text{-}20}$ peptide, generated with AlphaFold-Multimer [1], and the crystal structure of the *E. coli* MinD dimer in complex with $MinE^{I24N}$ (PDB: 3R9J) [14]. The box provides a comparison of the helix H4/H6/H7 region of ParA with the corresponding region of MinD, with ParA residues involved in ParB binding highlighted in blue.

SUPPLEMENTARY TABLES

Supplementary Table 1. Crystallographic data collection and refinement statistics.

**His_6-$ParA_{21\text{-}274}$-D60A dimer in complex with ATP** (PDB: **8RAY**)	
**Data collection**	
Space group	$P2_12_12_1$
Cell dimensions	
a, *b*, *c* (Å)	59.74 85.78 97.28
α, β, γ (°)	90 90 90
Wavelength (Å)	0.885600
Resolution (Å)	37.72 - 1.582 (1.639 - 1.582)
R_{merge}	0.1363 (2.179)
$I / \sigma I$	12.99 (1.55)
Completeness (%)	98.32 (83.71)
Redundancy	13.2 (12.5)
CC1/2	0.999 (0.673)
**Refinement**	
Resolution (Å)	37.72 - 1.582 (1.61 - 1.582)
No. reflections	67711 (5682)
R_{work} / R_{free}	0.17/0.20
No. atoms	4354
Protein	3905
Ligand/ion	64
Water	385
B-factors	27.90
Protein	27.06
Ligand/ion	19.79
Water	37.72
R.m.s. deviations	
Bond lengths (Å)	0.016
Bond angles (°)	1.59
Ramachandran	
Favored (%)	98.01
Allowed (%)	1.79
Outliers (%)	0.2

Values in parentheses are for the highest-resolution shell.

Supplementary Table 2. Strains used in this study.

Strain	Genotype/description	Construction	Reference/Source
Myxococcus xanthus			
DK1622	*M. xanthus* wild-type strain		[15]
SA4269	DK1622 Δ*parB* P_{cuo}-*parB*	Integration of pAH57 in DK1622 and subsequent deletion of *parB* with pAH18	[16]
LS004	DK1622 Δ*parB* P_{cuoA}-*parB* P_{van}-*sfmTurq2ox-parB*$_{R13A}$	Integration of pLS007 in SA4269	This study
LS005	*DK1622* Δ*parB* P_{cuoA}-*parB* P_{van}-*sfmTurq2ox-parB*$_{R13K}$	Integration of pLS009 in SA4269	This study
LS007	*DK1622* Δ*parB* P_{cuoA}-*parB* P_{van}-*sfmTurq2ox-parB*$_{\Delta 21}$	Integration of pLS011 in SA4269	This study
LS014	*DK1622* Δ*parB* P_{cuoA}-*parB* P_{van}-*mNeongreen-parB*	Integration of pMO116 in SA4269	This study
LS015	*DK1622* Δ*parB* P_{cuoA}-*parB* P_{van}-*mNeongreen-parB*$_{\Delta 21}$	Integration of pLS016 in SA4269	This study
MO072	DK1622 Δ*parB* P_{cuoA}-*parB* P_{van}-*sfmTurq2ox-parB*	Integration of pMO115 in SA4269	[17]
Escherichia coli			
TOP10	F^- *mcr*A Δ(*mrr-hsd*RMS-*mcr*BC) Φ80*lac*ZΔM15 Δ*lac*X74 *rec*A1 *ara*D139 Δ(*ara leu*) 7697 *gal*U *gal*K *rps*L (Str^R) *end*A1 *nup*G	-	Invitrogen
Rosetta(DE3) pLysS	F^- *ompT hsdS*$_B$(r_B^- m_B^-) *gal dcm* (DE3) pLysSRARE (Cam^R)	-	Merck Millipore

Supplementary Table 3. Plasmids used in this study.

Plasmid	Description	Construction/reference
**Plasmids used for cloning purposes**		
pTB146		[18]
pMR3690		[19]
pET-45b(+)		Novagen (Cat. #: 71327)
**Plasmids constructed in this study**		
pAH17	pET-45b(+) bearing *parA*	[16]
pJHA021	pTB146 bearing *parB*$_{L11S\ Q52A}$	Site-directed mutagenesis of pMO139 with primers oJHA029 and oJHA030
pJHA022	pTB146 bearing *parB*$_{L15S\ Q52A}$	Site-directed mutagenesis of pMO139 with primers oJHA031 and oJHA032
pJHA023	pTB146 bearing *parB*$_{L18S\ Q52A}$	Site-directed mutagenesis of pMO139 with primers oJHA033 and oJHA034
pLS007	pMR3690 bearing *sfmTurq2ox-parB*$_{R13A}$	Site-directed mutagenesis of pMO115 with primers MO251 and MO252
pLS008	pTB146 bearing *parB*$_{R13K}$	Site-directed mutagenesis of pMO104 with primers LS013 and LS014
pLS009	pMR3690 bearing *sfmTurq2ox-parB*$_{R13K}$	Site-directed mutagenesis of pMO115 with primers LS013 and LS014
pLS011	pMR3690 bearing *sfmTurq2ox-parB*$_{\Delta 21}$	a) PCR amplification of *parB* $_{\Delta 21}$ from pMO104 with primers MO199 and LS017 and *sfmturq2ox* from pMO115 with primers MO196 and LS018<br>b) Insertion of the fragment into pMR3690 cut with NdeI and EcoRI by Gibson assembly
pLS016	pMR3690 bearing *mNeonGreen-parB*$_{\Delta 21}$	a) PCR amplification of *parB* $_{\Delta 21}$ from pMO104 with primers MO199 and LS017 and *mNeongreen* from pMO116 with primers MO196 and LS018<br>b) Insertion of the fragment into pMR3690 cut with NdeI and EcoRI by Gibson assembly
pLS021	pTB146 bearing *parB*$_{R13A\ Q52A}$	Site-directed mutagenesis of pMO142 with primers MO237 and MO238
pLS022	pTB146 bearing *parB*$_{R13K\ Q52A}$	Site-directed mutagenesis of pLS008 with primers MO237 and MO238
pLS023	pTB146 bearing *parB*$_{\Delta 21\ Q52A}$	Site-directed mutagenesis of pMO145 with primers MO237 and MO238
pLS027	pET-45b(+) bearing *parA*$_{Q190A\ R238E}$	Site-directed mutagenesis of pMO023 with primers LS038 and LS039
pLS028	pET-45b(+) bearing *parA*$_{L192\ R238E}$	Site-directed mutagenesis of pMO023 with primers LS040 and LS041
pLS029	pTB146 bearing *parB*$_{I19S\ Q52A}$	Site-directed mutagenesis of pMO142 with primers LS034 and LS035
pLS030	pET-45b(+) bearing *parA*$_{E82R}$	Site-directed mutagenesis of pAH17 with primers LS046 and LS047
pLS031	pET-45b(+) bearing *parA*$_{D110R}$	Site-directed mutagenesis of pAH17 with primers LS048 and LS049
pLS032	pET-45b(+) bearing *parA*$_{E117R}$	Site-directed mutagenesis of pAH17 with primers LS050 and LS051
pLS035	pET-45b(+) bearing *parA*$_{D110R\ E117R}$	Site-directed mutagenesis of pLS031 with primers LS050 and LS051
pLS036	pET-45b(+) bearing *parA*$_{Y81L}$	Site-directed mutagenesis of pAH17 with primers LS054 and LS055
pMO104	pTB146 bearing *parB*	[20]
pMO016	pET-45b(+) bearing *parA*$_{D60A}$	Site-directed 19utagénesis of pAH17 with primers MO270 and MO272
pMO023	pET-45b(+) bearing *parA*$_{R238E}$	a) PCR amplification of *parA*$_{R238E}$ from pMT325 with primers MO034 and MO035<br>b) Insertion of the fragment into pET-45b(+) cut with BamHI and HindIII by Gibson assembly
pMO115	pMR3690 bearing *sfmTurq2ox-parB*	[20]
pMO116	pMR3690 bearing *mNeonGreen-parB*	[20]
pMO139	pTB146 bearing *parB*$_{Q52A}$	[20]
pMO142	pTB146 bearing *parB*$_{R13A}$	Site-directed mutagenesis of pAH17 with primers MO251 and MO252
pMO145	pTB146 bearing *parB*$_{\Delta 21}$	Site-directed mutagenesis of pMO104 with primers MO246 and MO247
pMO184	pTB146 bearing *parA*$_{21\text{-}274\ D60A}$	a) PCR amplification of *parA*$_{21\text{-}274\ D60A}$ from pMO016 with primers LS029 and LS030<br>b) Insertion of the fragment into pTB146 cut with BamHI and SapI by Gibson assembly
pMTh005	pET-45b(+) bearing *parA*$_{L150S\ R238E}$	Site-directed mutagenesis of pMO023 with primers MTh009 and MTh010
pMTh006	pET-45b(+) bearing *parA*$_{L155S\ R238E}$	Site-directed mutagenesis of pMO023 with primers MTh011 and MTh012
pMTh007	pET-45b(+) bearing *parA*$_{L187S\ R238E}$	Site-directed mutagenesis of pMO023 with primers MTh013 and MTh014
pMTh012	pET-45b(+) bearing *parA*$_{V116S\ R238E}$	Site-directed mutagenesis of pMO023 with primers MTh017 and MTh018
pMTh013	pET-45b(+) bearing *parA*$_{L152S\ R238E}$	Site-directed mutagenesis of pMO023 with primers MTh015 and MTh016
pMTh014	pET-45b(+) bearing *parA*$_{L150S\ L155S\ R238E}$	Site-directed mutagenesis of pMTh005 with primers MTh011 and MTh012
pMTh015	pET-45b(+) bearing *parA*$_{V116S\ L150S\ R238E}$	Site-directed mutagenesis of pMTh005 with primers MTh017 and MTh018
pMTh020	pET-45b(+) bearing *parA*$_{L150S\ L152S\ R238E}$	Site-directed mutagenesis of pMO023 with primers MTh019 and MTh020

Supplementary Table 4. **Oligonucleotides used in this study.**

**Oligonucleotide**	**Sequence** (5' to 3')
BioTEG-*parS*-3-for	biotin-triethylene glycol-CCGGTGGAGCACTCACCACTCC
BioTEG-*parS*-3-rev	biotin-triethylene glycol-TCCGCTTGGCGTGAGTTCCTGACG
LS001	CCGGTGGAGCACTCACCACTCC
LS002	TCCGCTTGGCGTGAGTTCCTGACG
LS013	CGGGCCCTGGGGAAAGGGCTGTCCGCCC
LS014	GGGCGGACAGCCCTTTCCCCAGGGCCCGC
LS017	CGGATCCGGAGGCGGAACGCAGGCGGGCGCCACCGGGG
LS018	CGGCCCCGGTGGCGCCCGCCTGCGTTCCGCCTCCGGATCCGCC
LS029	CCACCATCACGTGGGTACCGGTGTGGGTCGTATCATCTGCA
LS030	ACTCGAGTGCGGCCGCAAGCTTTCAGGTGTCCCGCTTCATCAG
LS034	GTCCGCCCTCAACCCCCAGGCG
LS035	CGCCTGGGGGTTGAGGGCGGACAG
LS038	CACCATCGACCTGGTGAAGGCGGGCCTCAACCCGG
LS039	CCGGGTTGAGGCCCGCCTTCACCAGGTCGATGG
LS040	CCTGGTGAAGCAGGGCTCCAACCCGGACCTGAAG
LS041	CTTCAGGTCCGGGTTGGAGCCCTGCTTCACCAG
LS046	CCGGCACCATCTACAGAGCGCTGCTCAATG
LS047	CATTGAGCAGCGCTCTGTAGATGGTGCCG
LS048	CGCCACGCCGAGGCTCACCGGCGCCGAG
LS049	CGCCGGTGAGCCTCGGCGTGGCGGGCAC
LS050	CCGGCGCCGAGGTCAGGCTGGTCAACC
LS051	GGTTGACCAGCCTGACCTCGGCGCCG
LS054	CCGGCACCATCTTGGAAGCGCTGCTCAATG
LS055	GAGCAGCGCTTCCAAGATGGTGCCGGTG
parS-Mxan-wt	GAGGCTTGTTCCACGTGGAACGTCGGTTTTTCGGACGTTCCACGTGGAACAAGC
MO034	GCGGGATCCCGTGCACTGCATCACGCGC
MO035	GCCAAGCTTTCATCAAGCCACGCGCCTGCG
MO196	CACGATGCGAGGAAACGCATATGGTGAGCAAGGGCGAGGAG
MO199	TACGCGTAACGTTCGAATTCCTACTCCTTCCTGAGAAGCTTCAAG
MO246	AGAACAGATTGGTGGTCAGGCGGGCGCCACCGG
MO247	CGGTGGCGCCCGCCTGACCACCAATCTGTTCTCT
MO251	GGGCCCTGGGGGCCGGGCTGTCCG
MO252	GCGGACAGCCCGGCCCCCAGGGCCC
MO270	CTGGTGGACATGGCCCCGCAGGGCAAC
MO272	GCGTTGCCCTGCGGGGCCATGTCCACC
MTh009	CTGTCCGCCGTCGAGCGGCCTGCTGACG
MTh010	GCGTCAGCAGGCCGCTCGACGGCGGAC
MTh011	CGGCCTGCTGACGAGCAATGCGCTGGCC
MTh012	GGCCAGCGCATTGCTCGTCAGCAGGCCG
MTh013	CCCACACCATCGACAGCGTGAAGCAGGGCCTC
MTh014	GAGGCCCTGCTTCACGCTGTCGATGGTGTGGG
MTh015	CGCCGTCGCTCGGCTCGCTGACGCTCAATG
MTh016	CATTGAGCGTCAGCGAGCCGAGCGACGGCG
MTh017	CACCGGCGCCGAGAGCGAGCTGGTCAAC
MTh018	GTTGACCAGCTCGCTCTCGGCGCCGGTG
MTh019	CATTGAGCGTCAGCGAGCCGGACGACGGCG
MTh020	CGCCGTCGTCCGGCTCGCTGACGCTCAATG
oJHA029	TCGGGGCGCGGGCTGTCCGCCCTC
oJHA030	GGCCCGCTTCTGCATGTCTGCTTTCACCAC
oJHA031	TCGTCCGCCCTCATCCCCCAGGCGG
oJHA032	CCCGCGCCCCAGGGCCCGCTTCTG
oJHA033	AGCATCCCCCAGGCGGGCGCCAC
oJHA034	GGCGGACAGCCCGCGCCCCAGGG

SUPPLEMENTARY REFERENCES

1. Evans R., *et al.* Protein complex prediction with AlphaFold-Multimer.). *bioRxiv* **DOI:** 10.1101/2021.10.04.463034 (2022).
2. Chu C. H., *et al.* Crystal structures of *Hp*Soj-DNA complexes and the nucleoid-adaptor complex formation in chromosome segregation. *Nucleic Acids Res.* **47**, 2113-2129 (2019).
3. Bartosik A. A., *et al.* Dissection of the region of *Pseudomonas aeruginosa* ParA that is important for dimerization and interactions with its partner ParB. *Microbiology (Reading)* **160**, 2406-2420 (2014).
4. Chu C. H., *et al.* Insights into the molecular mechanism of ParAB*S* system in chromosome partition by *Hp*ParA and *Hp*ParB. *Nucleic Acids Res.* **52**, 7321-7336 (2024).
5. Volante A. & Alonso J. C. Molecular anatomy of ParA-ParA and ParA-ParB interactions during plasmid partitioning. *J. Biol. Chem.* **290**, 18782-18795 (2015).
6. Zhang H. & Schumacher M. A. Structures of partition protein ParA with nonspecific DNA and ParB effector reveal molecular insights into principles governing Walker-box DNA segregation. *Genes Dev.* **31**, 481-492 (2017).
7. Leonard T. A., Butler P. J. & Löwe J. Bacterial chromosome segregation – structure and DNA binding of the Soj dimer – a conserved biological switch. *EMBO J.* **24**, 270-282 (2005).
8. Parker A. V., Mann D., Tzokov S. B., Hwang L. C. & Bergeron J. R. C. The structure of the bacterial DNA segregation ATPase filament reveals the conformational plasticity of ParA upon DNA binding. *Nat. Commun.* **12**, 5166 (2021).
9. Pratto F., *et al. Streptococcus pyogenes* pSM19035 requires dynamic assembly of ATP-bound ParA and ParB on *parS* DNA during plasmid segregation. *Nucleic Acids Res.* **36**, 3676-3689 (2008).
10. Schumacher M. A., *et al.* Structural mechanism of ATP-induced polymerization of the partition factor ParF: implications for DNA segregation. *J. Biol. Chem.* **287**, 26146-26154 (2012).
11. Yen C. Y., *et al.* Chromosome segregation in Archaea: SegA- and SegB-DNA complex structures provide insights into segrosome assembly. *Nucleic Acids Res.* **49**, 13150-13164 (2021).
12. Schumacher M. A., *et al.* Structures of archaeal DNA segregation machinery reveal bacterial and eukaryotic linkages. *Science* **349**, 1120-1124 (2015).
13. Wu W., Park K. T., Holyoak T. & Lutkenhaus J. Determination of the structure of the MinD-ATP complex reveals the orientation of MinD on the membrane and the relative location of the binding sites for MinE and MinC. *Mol. Microbiol.* **79**, 1515-1528 (2011).
14. Park K. T., *et al.* The Min oscillator uses MinD-dependent conformational changes in MinE to spatially regulate cytokinesis. *Cell* **146**, 396-407 (2011).
15. Kaiser D. Social gliding is correlated with the presence of pili in *Myxococcus xanthus*. *Proc. Natl. Acad. Sci. U S A* **76**, 5952-5956 (1979).
16. Harms A., Treuner-Lange A., Schumacher D. & Søgaard-Andersen L. Tracking of chromosome and replisome dynamics in *Myxococcus xanthus* reveals a novel chromosome arrangement. *PLoS Genet.* **9**, e1003802 (2013).
17. Osorio-Valeriano M., *et al.* ParB-type DNA segregation proteins are CTP-dependent molecular switches. *Cell* **179**, 1512-1524 (2019).
18. Bendezu F. O., Hale C. A., Bernhardt T. G. & de Boer P. A. RodZ (YfgA) is required for proper assembly of the MreB actin cytoskeleton and cell shape in *E. coli*. *EMBO J.* **28**, 193-204 (2009).
19. Iniesta A. A., Garcia-Heras F., Abellon-Ruiz J., Gallego-Garcia A. & Elias-Arnanz M. Two systems for conditional gene expression in *Myxococcus xanthus* inducible by isopropyl-b-D-thiogalactopyranoside or vanillate. *J. Bacteriol.* **194**, 5875-5885 (2012).
20. Osorio-Valeriano M., *et al.* The CTPase activity of ParB determines the size and dynamics of prokaryotic DNA partition complexes. *Mol. Cell* **81**, 3992-4007 (2021).
