# Supplementary material for: Molecular basis of ParA ATPase activation by the CTPase ParB during bacterial chromosome segregation
Source: Nat Commun. 2025 Sep 25;16:8428. doi: 10.1038/s41467-025-63976-0 (PMC12462528; doi:10.1038/s41467-025-63976-0)
Supplement: Supplementary file 2 — Reporting Summary [file 41467_2025_63976_MOESM2_ESM.pdf]

nature portfolio

Corresponding author(s): Martin Thanbichler

Last updated by author(s): Jul 3, 2025

Reporting Summary

Nature Portfolio wishes to improve the reproducibility of the work that we publish. This form provides structure for consistency and transparency in reporting. For further information on Nature Portfolio policies, see our Editorial Policies and the Editorial Policy Checklist.

Statistics

For all statistical analyses, confirm that the following items are present in the figure legend, table legend, main text, or Methods section.

n/a	Confirmed	
[ ]	[x]	The exact sample size (*n*) for each experimental group/condition, given as a discrete number and unit of measurement
[ ]	[x]	A statement on whether measurements were taken from distinct samples or whether the same sample was measured repeatedly
[x]	[ ]	The statistical test(s) used AND whether they are one- or two-sided<br>*Only common tests should be described solely by name; describe more complex techniques in the Methods section.*
[x]	[ ]	A description of all covariates tested
[x]	[ ]	A description of any assumptions or corrections, such as tests of normality and adjustment for multiple comparisons
[ ]	[x]	A full description of the statistical parameters including central tendency (e.g. means) or other basic estimates (e.g. regression coefficient) AND variation (e.g. standard deviation) or associated estimates of uncertainty (e.g. confidence intervals)
[x]	[ ]	For null hypothesis testing, the test statistic (e.g. *F*, *t*, *r*) with confidence intervals, effect sizes, degrees of freedom and *P* value noted<br>*Give P values as exact values whenever suitable.*
[x]	[ ]	For Bayesian analysis, information on the choice of priors and Markov chain Monte Carlo settings
[x]	[ ]	For hierarchical and complex designs, identification of the appropriate level for tests and full reporting of outcomes
[x]	[ ]	Estimates of effect sizes (e.g. Cohen's *d*, Pearson's *r*), indicating how they were calculated

Our web collection on statistics for biologists contains articles on many of the points above.

Software and code

Policy information about availability of computer code

Data collection	AlphaFold2/AlphaFold-Multimer (colab.research.google.com/github/sokrypton/ColabFold/blob/main/AlphaFold2.ipynb); AlphaFold 3 (https://alphafoldserver.com/); BLAST (blast.ncbi.nlm.nih.gov/Blast.cgi); BLItz Pro 1.2.1.5 (ForteBio Inc., USA); Gen5 2.07.17 (BioTek, USA); Image Lab (BioRad, Germany); VisiView 4.0.0.14 (Visitron Systems, Germany)
Data analysis	Adobe Illustrator CS6 (https://www.adobe.com/products/illustrator.html); BacStalk (https://drescherlab.org/data/bacstalk); CARA (http://cara.nmr.ch/doku.php/home); ChimeraX 1.5 (https://www.rbvi.ucsf.edu/chimerax); Clustal Omega (http://www.clustal.org/omega); Coot (https://www2.mrc-lmb.cam.ac.uk/personal/pemsley/coot/); DynamX 3.0.0 (https://www.waters.com/waters/library.htm?cid=511436&lid=134832928&locale=en_US); Fiji 1.49 (https://fiji.sc); MATLAB (https://www.mathworks.com); Jalview (https://www.jalview.org/); Microsoft Excel 2016 and Excel 365 (https://www.microsoft.com/microsoft-365); NMRFAM-Sparky (https://www.cgl.ucsf.edu/home/sparky/); PHASER (https://www-structmed.cimr.cam.ac.uk/phaser_obsolete/); Phenix 1.19.1 (https://phenix-online.org/); ProteinLynx Global SERVER version 3.0.1 (https://www.waters.com/waters/en_US/ProteinLynx-Global-SERVER-(PLGS)/nav.htm?cid=513821&locale=en_US); UCSF Weblogo 2.8.2 (https://weblogo.berkeley.edu/); XDS (https://xds.mr.mpg.de/); XSCALE (https://xds.mr.mpg.de/html_doc/xscale_program.html);

For manuscripts utilizing custom algorithms or software that are central to the research but not yet described in published literature, software must be made available to editors and reviewers. We strongly encourage code deposition in a community repository (e.g. GitHub). See the Nature Portfolio guidelines for submitting code & software for further information.

Data

Policy information about availability of data

All manuscripts must include a data availability statement. This statement should provide the following information, where applicable:

- Accession codes, unique identifiers, or web links for publicly available datasets
- A description of any restrictions on data availability
- For clinical datasets or third party data, please ensure that the statement adheres to our policy

The coordinates and structure factors for the crystal structure of the His6-ParA21-274-D60A•ATP dimer were deposited at the RCSB Protein Data Bank (PDB) under the accession code 8RAY. The PDB accession codes for the published crystal structures referenced in this study are: 6IUD, 2BEK, 7NPD, 2OZE, 4E09, 7DV3, 5K5Z, 3Q9L and 3R9J. The assigned NMR chemical shifts of His6-ParA21-274-D60A have been deposited to the Biological Magnetic Resonance Data Bank (bmrb.io) under accession number 52940. HDX-MS data have been deposited to the ProteomeXchange Consortium via the PRIDE partner repository under the dataset identifier PXD063735. All other data supporting the findings of this study are included in the main text or the supplementary material. Source data are provided with this paper.

Research involving human participants, their data, or biological material

Policy information about studies with human participants or human data. See also policy information about sex, gender (identity/presentation), and sexual orientation and race, ethnicity and racism.

Reporting on sex and gender	Not applicable.
Reporting on race, ethnicity, or other socially relevant groupings	Not applicable.
Population characteristics	Not applicable.
Recruitment	Not applicable.
Ethics oversight	Not applicable.

Note that full information on the approval of the study protocol must also be provided in the manuscript.

Field-specific reporting

Please select the one below that is the best fit for your research. If you are not sure, read the appropriate sections before making your selection.

- [x] Life sciences
- [ ] Behavioural & social sciences
- [ ] Ecological, evolutionary & environmental sciences

For a reference copy of the document with all sections, see nature.com/documents/nr-reporting-summary-flat.pdf

Life sciences study design

All studies must disclose on these points even when the disclosure is negative.

Sample size	At least 300 cells were analyzed per strain to quantify the number and distribution of partition complexes. At least 30 cells were analyzed per strain in the FRAP experiments. In our experience, this sample size is sufficient to allow robust measurements of the parameters analyzed.
Data exclusions	No data were excluded from the analyses.
Replication	We routinely analyzed multiple independent strains to verify the phenotypes observed. All experiments were performed at least twice to ensure reproducibility, and similar results were obtained throughout.
Randomization	All separable cells in the microscopy images were analyzed. The images were selected randomly. Similarly, random cells were analyzed in the FRAP experiments.
Blinding	Data analysis were not performed blindly to facilitate data storage and analysis. However, care was taken not to introduce any bias during data acquisition and analysis.

Reporting for specific materials, systems and methods

We require information from authors about some types of materials, experimental systems and methods used in many studies. Here, indicate whether each material, system or method listed is relevant to your study. If you are not sure if a list item applies to your research, read the appropriate section before selecting a response.

Materials & experimental systems

n/a	Involved in the study	
[ ]	[x]	Antibodies
[x]	[ ]	Eukaryotic cell lines
[x]	[ ]	Palaeontology and archaeology
[x]	[ ]	Animals and other organisms
[x]	[ ]	Clinical data
[x]	[ ]	Dual use research of concern
[x]	[ ]	Plants

Methods

n/a	Involved in the study	
[x]	[ ]	ChIP-seq
[x]	[ ]	Flow cytometry
[x]	[ ]	MRI-based neuroimaging

Antibodies

Antibodies used	A polyclonal anti-GFP antibody (Sigma-Aldrich, Cat# G1544, RRID:AB_439690) was used at dilutions of 1:10,000.
Validation	The specificity of the antibody was verified by immunoblot analysis of reference strains producing known GFP-tagged proteins.

Plants

Seed stocks	Not applicable.
Novel plant genotypes	Not applicable.
Authentication	Not applicable.
